# OCT-Guided Surgery for Gliomas: Current Concept and Future Perspectives

**DOI:** 10.3390/diagnostics12020335

**Published:** 2022-01-28

**Authors:** Konstantin Yashin, Matteo Mario Bonsanto, Ksenia Achkasova, Anna Zolotova, Al-Madhaji Wael, Elena Kiseleva, Alexander Moiseev, Igor Medyanik, Leonid Kravets, Robert Huber, Ralf Brinkmann, Natalia Gladkova

**Affiliations:** 1 Department of Oncology and Neurosurgery, University Clinic, Privolzhsky Research Medical University, 18/1 Verkhnevolzhskaya Naberezhnaja, 603155 Nizhny Novgorod, Russia; zolotova_Anna.1997@mail.ru (A.Z.); waelawn44@gmail.com (A.-M.W.); med_neuro@inbox.ru (I.M.); l.ya.kravetc@gmail.com (L.K.); 2 Institute of Biomedical Optics, University of Luebeck, Peter-Monnik-Weg 4, D-23562 Luebeck, Germany; bonsanto@me.com (M.M.B.); robert.huber@uni-Luebeck.de (R.H.); ralf.brinkmann@uni-luebeck.de (R.B.); 3 Institute of Experimental Oncology and Biomedical Technologies, Privolzhsky Research Medical University, 10/1 Minin and Pozharsky Sq., 603950 Nizhny Novgorod, Russia; achkasova.k@bk.ru (K.A.); kiseleva84@gmail.ru (E.K.); natalia.gladkova@gmail.com (N.G.); 4 Institute of Applied Physics of the Russian Academy of Sciences, 46 Ulyanova St., 603950 Nizhny Novgorod, Russia; Aleksandr.Moiseev@gmail.com

**Keywords:** optical coherence tomography, brain imaging, neurosurgical guidance, brain tumor, minimally invasive theranostics, intraoperative imaging

## Abstract

Optical coherence tomography (OCT) has been recently suggested as a promising method to obtain in vivo and real-time high-resolution images of tissue structure in brain tumor surgery. This review focuses on the basics of OCT imaging, types of OCT images and currently suggested OCT scanner devices and the results of their application in neurosurgery. OCT can assist in achieving intraoperative precision identification of tumor infiltration within surrounding brain parenchyma by using qualitative or quantitative OCT image analysis of scanned tissue. OCT is able to identify tumorous tissue and blood vessels detection during stereotactic biopsy procedures. The combination of OCT with traditional imaging such as MRI, ultrasound and 5-ALA fluorescence has the potential to increase the safety and accuracy of the resection. OCT can improve the extent of resection by offering the direct visualization of tumor with cellular resolution when using microscopic OCT contact probes. The theranostic implementation of OCT as a part of intelligent optical diagnosis and automated lesion localization and ablation could achieve high precision, automation and intelligence in brain tumor surgery. We present this review for the increase of knowledge and formation of critical opinion in the field of OCT implementation in brain tumor surgery.

## 1. Introduction

Malignant gliomas are the most common brain tumors and account for 63% of all astrocytic tumors [1]. Their distinctive trait is infiltrating growth into the surrounding white matter of the brain, making the boundary between the tumor and brain tissue difficult to discern. Prognosis in this group of patients is largely determined by the morphological and molecular genetic characteristics of the tumor. Astrocytic tumors are conventionally divided into slow-growing and fast-growing ones in terms of growth rate and degree of invasion. The first are the pilocytic astrocytoma (Grade 1) and diffuse astrocytoma (Grade 2), the second are anaplastic astrocytoma (Grade 3) and glioblastoma (Grade 4) [2]. The average life expectancy for anaplastic astrocytoma and glioblastoma is 25 and 14 months, respectively [1]. In the case of low-grade (1–2) astrocytomas, life expectancy in different studies ranges from 61.1 to 90 months [3]. For these groups, the astrocyte is important for malignant tumor transformation, that is, its transition to the malignant variant (Grade 3–4), which is observed in about 45% of patients within 5 years [4,5]. 

The removal of a tumor is an important stage of treatment, as it allows the extraction of a large volume of tumor tissue, the reduction of intracranial pressure, the decrease of neurological deficit and the establishment of an exact tumor phenotype to solve the question of further treatment tactics. Modern research has shown that the size of tumor resection is reliably correlated with the life expectancy of patients [6,7,8,9,10]. In the case of diffuse astrocytomas with a low degree of malignancy, total tumor removal is a key prognostic factor as it effectively reduces the risk of malignant tumor transformation [3].

The main paradigm of glial tumor surgery is the maximal removal of the tumor with minimal risk of damage to functionally significant areas of the brain [11,12,13,14]. However, the extraction of a tumor in the white light of a microscope can only achieve a maximum resection in 23–50% of cases [8,9,15]. This contributes to maintaining a high interest in the development of intraoperative technologies that allow the differentiation of tumors from surrounding tissues. Currently all intraoperative diagnostic methods are based on several approaches:

- Determination of contrast agents accumulating in the tumor vascular net (CT, MRI with contrast);
- Determination of metabolic changes in tissues (5-ALA fluorescence, laser spectroscopy);
- Determination of brain areas with altered blood–brain barriers with fluorescein;
- Determination of changes in tissue density (ultrasound).

Intraoperative magnetic resonance imaging (iMRT) and fluorescence diagnostics have been found to be most effective methods. However, there are several limitations to the use of these methods. A number of researchers have indicated low sensitivity of fluorescent diagnostics for low-grade malignant glioma [16,17]. Moreover, the assessment of fluorescence intensity is conducted by the surgeon and is therefore subjective, which can result in the preservation of a part of the tumor tissue possessing low fluorescence [18]. iMRT has a number of disadvantages, including high cost, inability to integrate with the microscope and the need to use special surgical tools. The technique has a long learning curve and requires a high level of training for surgeons [19].

The lack of a «perfect» method of intraoperative visualization determines the relevance of finding other efficient technologies, among which optical coherence tomography (OCT) looks attractive. OCT imaging has some benefits in comparison with other intraoperative technologies in neurosurgery: high resolution, high speed, low cost, no need to use contrast agents, non-invasiveness and convenient performance. Currently, OCT is available for intraoperative use in neurosurgery as a special microscope module [20,21], or using various optical probes [22,23]. 

Among the entire spectrum of OCT modalities, the effectiveness of OCT for the differentiation of tumor and white matter based on the analysis of structural images is experimentally demonstrated [24,25,26]. In addition, OCT may be used to determine the extent of white matter damage at the edge of tumor resection. Clinical trials for intraoperative tissue discrimination are in progress. It is necessary to mention that the potential of OCT does not appear to be limited to neuro-oncology [27,28]. A number of studies demonstrate the effectiveness of OCT in the visualization of peripheral nerves [29,30] and some research has been conducted on the use of OCT in vascular and functional neurosurgery. 

In this paper, we will focus on main types of OCT images obtained in the brain and the results of their application in practical neurosurgery. We present this review for the increase of knowledge and formation of critical opinion in the field of OCT implementation in brain tumor surgery.

## 2. Materials and Methods

“Pub Med”, “Cochrane Library”, “SPIE Digital Library” and “IEEE Xplore” databases were screened for “optical coherence tomography + brain tumors”, “optical coherence tomography + intraoperative imaging + brain tumors” and “optical coherence tomography + neurosurgery”, “intraoperative imaging + brain tumors”. Detailed evaluation of the results revealed 12 articles related to the use of OCT for ex vivo and in vivo visualization of brain tumors. N = 6 articles documented the use of OCT for distinguishing tumorous and non-tumorous tissue in astrocytoma. 

## 3. OCT Multimodality and Multitasking

### 3.1. OCT Multimodality

Active introduction of OCT into clinical practice is determined by clear benefits of this method with no risk of tissue damage, high resolution (~10 micron), absence of necessity of contrast agents, imaging depths of more than 1 mm, acquisition of images in a contactless way and therefore availability to be integrated into surgical microscope or endoscope [31]. 

Moreover, compared to other intraoperative technologies, OCT can provide a variety of information for tissue function based on so-called functional OCT. There are the following extensions: Doppler OCT (DOCT) and OCT angiography (OCTA), spectroscopic OCT (SOCT) and molecular imaging OCT [32]. Some of them look promising for clinical application in brain tumor surgery. 

***Polarization-sensitive (PS) OCT or cross-polarization (CP) OCT***

Conventional, intensity-based OCT has demonstrated impressive results in detecting any pathological changes of stratified tissues, such as the eye. However, for the advanced visualization of structureless tissue types (brain, breast) the PS (CP) OCT looks to be the more promising method. The main feature of PS OCT is the ability to detect the polarization state changes of the probing light in the tissue, thereby generating the tissue-specific contrast [33,34]. Based on the birefringence of the tissue structure, PS OCT provides better visualization of elongated structures and therefore allows advanced imaging of myelinated nerve fibers in nerves and brain [35,36]; even the orientation of white matter tracts can be detected [37,38]. CP OCT is the variation of PS OCT, which allows imaging of initial polarization state changes both due to birefringence and cross-scattering in biological tissue [39,40]. The CP OCT imaging has demonstrated higher diagnostic accuracy in distinguishing tumorous tissue and white matter in comparison with conventional OCT imaging (87–88% compared to 83–84%, respectively) [22]. 

***Doppler OCT (DOCT) and OCT angiography (OCTA)*** are functional imaging techniques performing quantification of the speed of moving particles with high spatial resolution that allows building the structural imaging of the vessel network [41]. It is attractive for visualization of cerebral microvasculature due to short acquisition time, high spatial resolution, depth-resolved information, absolute flow measurement and non-invasiveness [41]. OCTA is widely used in preclinical cancer research, e.g., for studying tumor angiogenesis and the vascular drug response [28,42]. The OCTA visualization of cerebral and tumorous vessels has demonstrated great differences between them in form, density and size; the tumorous vessels are convoluted, with uneven contours to the lumen [25]. However, the benefits of OCTA clinical application for neurosurgeons are still unclear. 

### 3.2. OCT Application in Neurosurgery

There are several scenarios as to how OCT can be implemented in neurosurgery: (1) OCT can be used intraoperatively for brain imaging and can provide real-time feedback to the surgeons, e.g., clarifying the boundaries of the infiltrative brain tumors within surrounding tissues and the degree of white matter damage; (2) OCT can be used in histopathological studies of fresh specimens for fast determination of tissue type; (3) OCT can aid in stereotactic procedures for guiding biopsy. Possible solutions to each of the assigned tasks are discussed below, while Section 6 is devoted to the solution of the first problem, where main attention is paid to the delineation of tumorous and normal brain tissues. The use of OCT during stereotaxic biopsy is discussed in Section 7. 

## 4. Clinical OCT Devices

**Figure 1:**
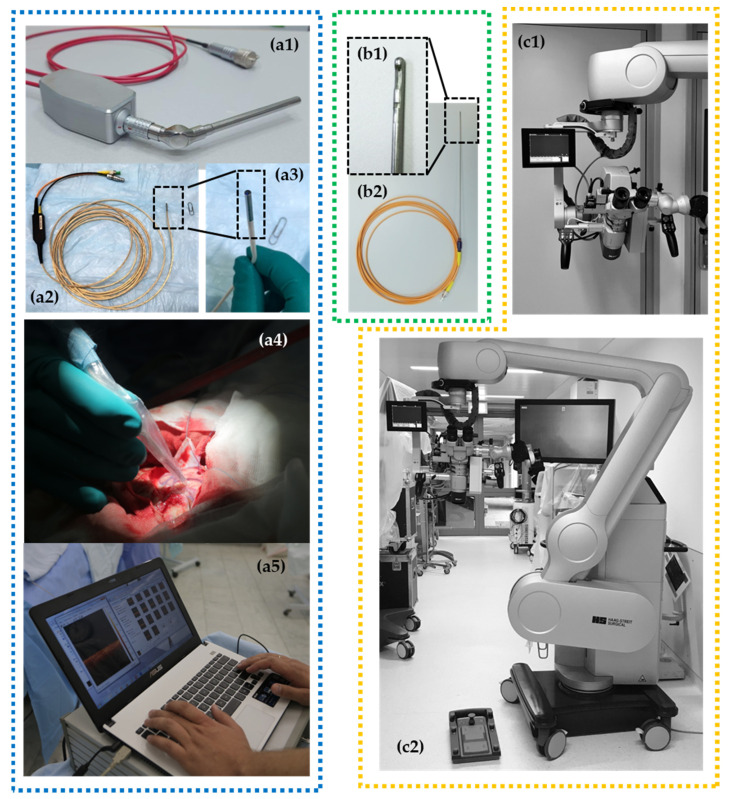
Different OCT devices are presented with corresponding types of OCT probes: (1) rigid curve-shaped (**a1**) and flexible (**a2**,**a3**) handheld probes for intraoperative use with portable OCT device (**а5**) and application during brain tumor removal (**a4**); (2) needle-like probe (**b1**,**b2**) for stereotactic biopsy providing 2D images; (3) operating microscope with integrated OCT system providing OCT imaging directly in oculars and on a small-sided screen* fixed close to the microscope ocular (**c1**,**c2**).

According to these possible implementations, several OCT scanner designs have been investigated (Figure 1): handheld imaging probes [22,23,24,36], surgical instrumentation (e.g., biopsy needle) [9,10,11], microscope-integrated systems [20,43,44] and non-portable stationary OCT systems [25,45,46] that can be used in histopathological studies as a part of hybrid optical imaging systems providing label-free histology [47]. Considering multifunctionality of OCT in neurosurgery, the multipurpose device with a particular set of OCT probes could be preferred. 

Currently, microscope integrated OCT is available for neurosurgery. For example, the surgical microscope HS Hi-R 5-1000iFG (Haag-Streit Surgical GmbH, Wedel, Germany) with an integrated OCT camera can be mentioned. Scans are displayed directly by an external monitor and a small head-up display in the field of view of the surgeon (Figure 1(с1,с2)). Future OCT technology may be more deeply integrated in digital microscope (or exoscope) devices. This will offer the ability to integrate intraoperative image modalities such as structural MRI, functional MRI, tractography, fluorescence-guided surgery, intraoperative navigation and OCT in real-time [48].

Where the imaging probes are concerned, we have discovered that flexible handheld OCT probes of a small diameter are suitable for OCT-guided neurosurgery. The flexible handheld CP OCT probe was originally designed for endoscopic use at early stages of common-path OCT development. The 5 m long fiber probe was realized in a few modifications with outer diameter of scanning head varying from 1.6 mm to 2.7 mm. The probing beam driving mechanism was described in detail in [25]. The probe head design afforded compatibility with most commercially available endoscopes, but it may also be used independently. The small head size makes it possible to use the probe in any environment and previous studies have demonstrated its applicability for tumorous tissue detection during brain cancer resection using described qualitative (visual) CP OCT criteria [22]. However, the positioning of the probe can face several problems due to its flexibility. Moreover, if one needs enough scanning pattern stability for realization of color-coded maps, OCT angiography or elastography modalities that look promising for application during neurosurgical procedures, it is impossible to use this probe due to inability of the 3D scanning mode. 

The multimodal OCT imaging including calculation of optical coefficients and building color-coded maps can be realized by the rigid handheld probes which are the variants of dismountable probes.

OCT integration into the surgical microscope looks to be the most obvious and comfortable option for surgeons [20,43,44]. Realization of this idea requires the reconstruction of production-release design and building of so-called “OCT-ready” surgical microscopes by the developer company. Moreover, there are some challenges of large-scale/wide-field scanning of the resection zone: creating a map of the cavity, scanning of the perpendicular surface and merging an intraoperative visualization of the three-dimensional data [49].

The handheld imaging probes [22,23,24,36] do not need such technical advances and can be used jointly with the microscope for intraoperative assessment of tissues. In brain tumor surgery, OCT can be used for in situ detection of cancer tissue via “optical biopsy”. However, most of the suggested systems are not comfortable in clinical use due to their size, shape, excessive flexibility, etc. The bayonet-shaped OCT probe presented in this paper seems to be the most favorable option since this construction is familiar to the surgeon and preserves the field of view. 

## 5. Evaluation of OCT Data Obtained in Brain Tumor Surgery

**Figure 2:**
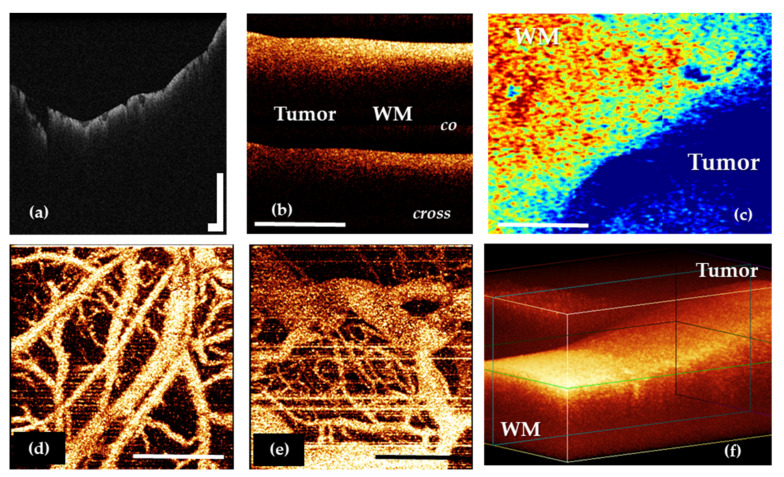
OCT data types in neuro-oncology: 2D OCT images of brain tissue obtained by OCT-integrated microscope (**a**) and portable CP OCT system with handled probe (**b**); based on attenuation coefficient color-coded map of border between white matter and glioma obtained by CP OCT device from human biopsy sample (**c**); OCTA images of cortex (**d**) and glioblastoma 101.6 in rat (**e**); 3D OCT image of border between white matter and glioma (**f**). WM—white matter. Scale bar is 1 mm in all images.

Regardless of the task that is solved in neurosurgery, OCT imaging may include several steps. The initial OCT data are obtained as 2D and 3D structural and angiographic pictures (Figure 2a,b,d–f), that can be visually analyzed. However, their more obvious presentation often requires quantitative assessment through optical coefficients calculation and construction of optical maps in a pseudo-color palette based on their values distribution (Figure 2c).

***Two-dimensional structural image*** analysis is the traditional method of estimating the OCT signal [20,22] that showed fairly high sensitivity (82–85%) and specificity (92–94%) in differentiation of white matter and glial brain tumors [22]. This approach is characterized by the following advantages: high speed of image acquisition, the possibility of rapid interpretation by the neurosurgeon and the availability of use in the operating room both by the module of the operating microscope and by the optical probe [20,22]. At the same time, visual analysis is not lacking in limitations such as low contrast of OCT images and the subjectivity of the surgical evaluation due to the requirement of the surgeon for perfect «reading» of OCT images and passing the corresponding learning curve. Unfortunately, use of this approach to evaluate OCT data does not meet the requirements of modern intraoperative imaging. 

Among all the types of OCT structural images, it is particularly worth highlighting the angiographic images, which are widely used in basic research in oncology and neuroscience [50,51,52,53]. This image modality may offer perspective during brain tumor removal. A number of studies performed on the experimental tumor models have shown that OCT allows the revealing of differences in the vascular architectonics of the cortex and the tumor (Figure 2d,e) [53,54]. The vasculature of the tumor tissue mainly consists of blood vessels of the same diameter; the vessels do not have a distinct direction, they are located chaotically and tightly. In shape, the tumor vessels are predominantly convoluted; the lumen of the vessels has uneven contours. At the same time, the cortex is characterized by the main type of branching, the presence of vessels of different diameters and, in general, a distinct direction of the vessels [25].

The ***calculation of optical coefficients*** is an approach that provides an objective assessment of the OCT of the data. The attenuation coefficient is the most common [20,24,55]; however, for OCT supporting polarization mode, other coefficients may be calculated depending on the method of signal processing [46,56]. Such quantitative evaluation of OCT data allows increasing the diagnostic accuracy of OCT for differentiation of tumor and white matter to 100% by using the threshold values of optical coefficients [24,46]. Despite its high accuracy and objectivity, in the future, all calculated coefficients will require a clear visual intraoperative representation for the surgeon, adapted to his intraoperative needs. 

The most promising is the use of ***color-coded maps*** built based on the calculated values of optical coefficients, which allows one to combine the accuracy of the quantitative approach and the convenience of assessing high-contrast images [24,46]. Currently, active work is underway to introduce this approach into clinical practice [23]. 

OCT image analysis problems solved using ***machine learning methods*** can be conditionally reduced to two categories: classification problems, i.e., tasks of establishing a diagnosis based on a single OCT image [57,58] and segmentation tasks, i.e., selection of areas on OCT images corresponding to a particular state of the tissue. Examples of such a task are determining the boundaries of pathology [23,59,60], the task of isolating certain morphological structures [61], the task of isolating blood flow [62] or the task of isolating fluid accumulations [63]. To increase the quality of classification, these approaches are sometimes combined: at the first stage of the algorithm, the structures that affect the diagnosis are segmented; at the second stage, the original images and segmentation results are jointly analyzed [64,65]. A feature of OCT is that the tasks of segmentation and classification are often mixed. Thus, the task of segmenting the volume of OCT data in a top view is reduced to the task of classifying individual distributions of the OCT signal in depth [23,66,67].

One should note that the problems of OCT image segmentation can often be solved with other approaches to the analysis of OCT data. For example, the boundaries of brain tumors can be determined from attenuation coefficient maps, and blood flow microcirculation networks can be found by one of the many methods known to date. Machine learning methods make it possible to reduce the distribution of OCT data in several modalities to a single criterion [59]. Or, as in the case of determining blood flow, they allow obtaining the desired result in conditions where physical reasons do not allow the use of alternative methods [62].

## 6. Basics of OCT Signal Forming in Nervous Tissue

OCT (wavelength of 800–1500 nm) matches the “biological imaging window”, where the light absorption is minimal and the penetration depth is maximal, since the wavelength of the incident light does not correspond to the absorption band of the chromophores (substances that can absorb radiation energy within a certain wavelength range). Therefore, the scattering change at the interface of two media makes the most significant contribution to the forming of the OCT signal. The scattering properties of the tissue may differ significantly for different types of cells and the surrounding extracellular material. 

The optical properties of the white and grey matter of the brain are different: white brain matter has significantly higher backscattering and absorption coefficients [68,69,70]. The grey matter mainly consists of cells with weak scattering properties, while the major portion of the white matter is made mostly of myelinated fibers (70–95% of all fibers) [71] characterized by high backscattering [20,69,70]. Myelinated fibers are covered with a specific myelin sheath—a membrane structure formed by repeated winding of the processes of oligodendrocytes on the axon of a neuron. This sheath is rich in lipids and proteins and is characterized by a relatively low water content (about 40%) [72]. The degree of signal attenuation is inversely related to the water content and the lower water content is associated with a rapid attenuation of OCT signal [73,74]. Therefore it can be assumed that myelin is the main component affecting the attenuation of the OCT signal, respectively; the degree of destruction of myelin fibers and the density and orderliness of their arrangement will be reflected in the change in the characteristics of the obtained OCT images.

The morphology of the tumorous tissue is characterized by the random nature of cell structures, variance in cellular nuclei size, change in the refractive index of the nucleus—cytoplasm, vascular proliferation and areas of necrosis which alter the nature of backscattering from a tissue [75].

## 7. Clarifying the Boundaries of the Infiltrative Glioma Growth

As was mentioned above, to carry out high-quality resection it is extremely important to distinguish tumor mass and white matter as this allows performing maximum removal of the tumorous tissue and avoiding postoperative complications associated with damage to white matter tracts. Using OCT for this purpose requires establishment of clear criteria of OCT images specific for each tissue type. Regardless of the type of device and image assessment approach used, OCT has demonstrated high diagnostic accuracy (Table 1).

### 7.1. Using OCT for White Matter and Tumor Differentiation

**Figure 3:**
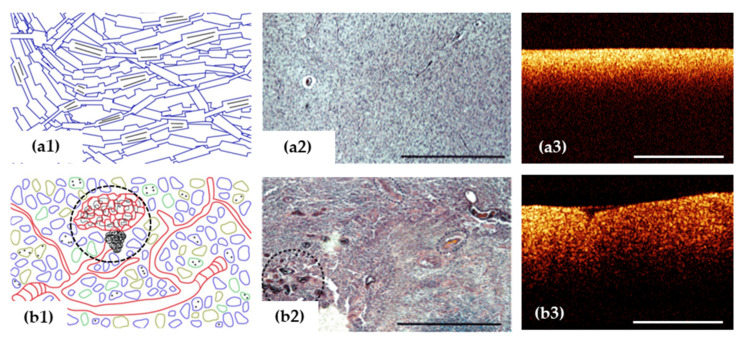
OCT signal (**a3**,**b3**) is formed by close-packed myelin fibers in white matter (**a1**–**a3**) and by a variety of cells, vascular proliferation and areas of necrosis (black dotted area) in glioblastoma demonstrated on histological images using hematoxylin and eosin staining (**b1**–**b3**). Scale bar is 1 mm in all images.

***Visual assessment*** criteria for white matter and tumor differentiation on two-dimensional OCT images were proposed in several studies [20,22]. Initial attempts have been made by various research groups to determine accurate criteria for the differential diagnosis of tumor and normal brain tissue according to the OCT signal character of intensity images (for multimodal OCT, this corresponds to the image in the co-polarization). Thus, Bohringer et al., based on qualitative analysis of OCT images of glial tumors of different malignancy (42 biopsies) identified the degree of signal homogeneity as a differential criterion, wherein the tumor tissue and the peritumoral area (infiltration zone) differed in the heterogeneous character of their signals, from the homogeneous character of the normal brain tissue [20,26]. A high degree of correlation between the character of the OCT signal and the histological findings was determined (χ^2^ test; r = 0.99) [20]. The OCT criteria for distinguishing tumorous tissue and white matter have been refined based on the results of study with CP OCT device [22]. Despite the variability of OCT images obtained from tumors, the main and additional features characteristic of all types of tumors were identified, which led to a high level of interrater agreement. In this case, it was proposed to use the intensity of the OCT signal in the co- and cross-polarization as the main criterion (Figure 3b), and as an additional factor, the homogeneity/heterogeneity of the signal and the uniformity of signal attenuation along the lower boundary in the co-polarization images [22].

Quantitative evaluation of OCT data based on the ***calculation of the attenuation coefficient*** allows performing differential diagnosis of a tumor and white matter with higher diagnostic accuracy in comparison with visual assessment of images. Additionally, optical maps reflecting the attenuation coefficient values distribution throughout the image provide a more contrasting image, which leads to better delineation between normal white matter and tumor. The attenuation coefficient was used for differentiation between tumor tissue and white matter for the first time by Kut et al. in 2015 [24]. They obtained representative value ranges for tumorous and normal brain tissues: for high malignancy gliomas core −3.9 ± 1.6 mm^−1^; for their infiltration zone −7.1 ± 1.0 mm^−1^; for low malignancy gliomas core −4.0 ± 1.4 mm^−1^; for their infiltration zone −2.7 ± 1.0 mm^−1^; and for normal white matter −6.2 ± 0.8 mm^−1^. To distinguish normal white matter and tumor tissue, a threshold value of attenuation coefficient of 5.5 mm^− 1^ was proposed, where high values of specificity and sensitivity were obtained (100% and 92% for high-grade patients, 80% and 100% for low-grade patients, respectively). Optical maps in a pseudo-color palette allowed visualizing the tumor, white matter and the border time.

However, it should be noted that the values of optical coefficients obtained in a number of studies differ from those presented in the paper of Kut et al., which can be explained by the differences in the used OCT devices and algorithms for quantitative processing of OCT data. For example, in the study [46], where both tumorous (low- and high-grade) and normal brain tissues were examined, the following values of the attenuation coefficient were collected: for normal white matter −8.5 [8.2; 9.3] mm^−1^; for diffuse astrocytoma (Grade 1–3) −3.0 [2.6; 3.5] mm^−1^; for glioblastoma (Grade 4) without necrotic areas −3.15 [2.6; 4.2] mm^− 1^; for glioblastoma (Grade 4) with necrotic areas −6.3 [5.4; 6.8] mm^− 1^; and for necrosis −7.5 [5.3; 7.7] mm^−1^. In accordance with the aforementioned numerical ranges, the threshold values for tumor/white matter differentiation were 8.2 mm^−1^ for patients of all grades (sensitivity/specificity: 95.6%/81.3%, respectively) and 6.1 mm^−1^ specifically for low-grade gliomas (sensitivity/specificity: 100%/100%, respectively) [46]. However, despite the variability in the results obtained by different groups, a similar tendency in the dependence between optical coefficients and structural features of the brain tissues may be noticed. 

Thus, the use of optical coefficients for neurosurgical guidance leads to the necessity to take into account the features of the OCT device and OCT data post-processing approach. Nevertheless, this method looks promising for in vivo studies of brain tissues in the operating room which was demonstrated in the paper by Almasian et al. [76], indicating the need to continue research in this area. They estimated the attenuation coefficient from OCT data collected from brain glioma and normal nervous tissue during tumor resection and reported the results as being compatible with previously published papers, indicating the relevance of using this approach for neuronavigation. Another intraoperative OCT study by Kut et al. has introduced a computational method for OCT-based automated detection of glioma infiltrated from non-cancerous brain tissue that demonstrated high detection accuracy (sensitivity/specificity: ~100% and ~85%, respectively), robustness and low computational cost of OCT-guided glioma resection [23]. However, the verification was performed using only ex vivo data. The in vivo data were collected on the model. The scans were not performed intraoperatively in vivo in a real resection cavity. Therefore, from our experience, the data regarding sensitivity and specificity should be viewed critically and this should also be presented as such.

White matter is characterized by high attenuation coefficients compared to tumorous tissue. However, in some cases, the decrease of white matter attenuation (brain edema, destruction of myelin) or increase of attenuation from tumorous tissue (necrosis) can cause difficulties in distinguishing between tumorous and non-tumorous tissue using both qualitative and quantitative approaches. Necrosis can occur in the following cases: (1) in the central parts of glioblastoma, (2) brain tissue after radiation therapy (for example, in the case of surgery for recurrent tumor growth) and (3) brain tissue after bipolar coagulation (total necrosis). Several studies have demonstrated that white matter attenuation can be reduced due to the destruction of myelin fibers or severe edema that usually appears in the peritumoral area of malignant infiltrating growing brain tumors [46,73,74]. Therefore, the diagnostic value of OCT is likely to decrease in the following cases: (1) resection of glioblastoma, where the edema in the peritumoral area is very prominent; (2) resection of continued tumor growth after combined treatment. 

### 7.2. Using OCT for Grey Matter and Tumor Differentiation

The distinguishing of tumorous tissue and grey matter is quite difficult using both qualitative and quantitative approaches due to the closely approximated OCT signal parameters [22,46,76]. The challenges in detection of tumor infiltration within grey matter and basal ganglia are not studied and discussed in most papers. The basal ganglia and thalamus are deep-seated structures of the brain and therefore present difficulties when performing OCT imaging. In the study of Jafri et al. the clear differences between white matter and grey matter of putamen, globus pallidum, thalamus and subthalamic nuclei were demonstrated [77]. Implementation of OCT will be difficult near the hippocampus, which has a lower scattering strength than the cortex [78].

**Figure 4:**
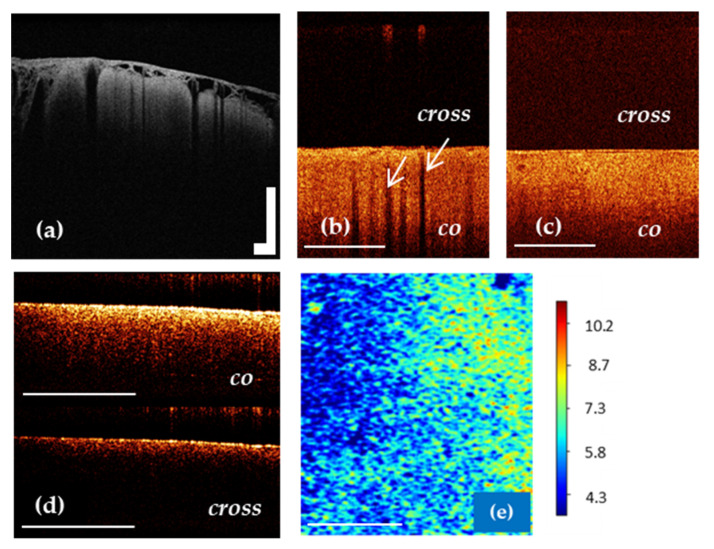
OCT imaging of cortex in vivo (**a**,**b**) using OCT-integrated microscope and handheld CP OCT probe, ex vivo (**d**) and color-coded OCT map of cortex based on the attenuation coefficient (**e**). The white arrows show a specific vertical striation arising from “shadows” of the blood vessels located just under the cortex surface (**b**), which disappear in the case of tumor infiltration of the cortex (**c**). The CP OCT image includes two parts: co-polarization (co) based on initial state of polarization after backscattering within the tissue and cross-polarization (cross) that determines any orthogonal polarization. Scale bar is 1 mm in all images.

It is known that in vivo OCT images of the cortex are characterized by specific vertical striation arising from “shadows” of the blood vessels located just under the tissue surface [22,79]. Therefore, the differentiation between cortex and tumorous tissue can be performed based on the criterion “loss of normal attenuation” (loss “typical view” with vertical striations on OCT images) (Figure 4b,c) [20,22]. The optical coefficients obtained from ex vivo specimens have not demonstrated significant differences between cortex and tumorous tissue [46,76]. 

### 7.3. OCT for White Matter State Evaluation

**Figure 5:**
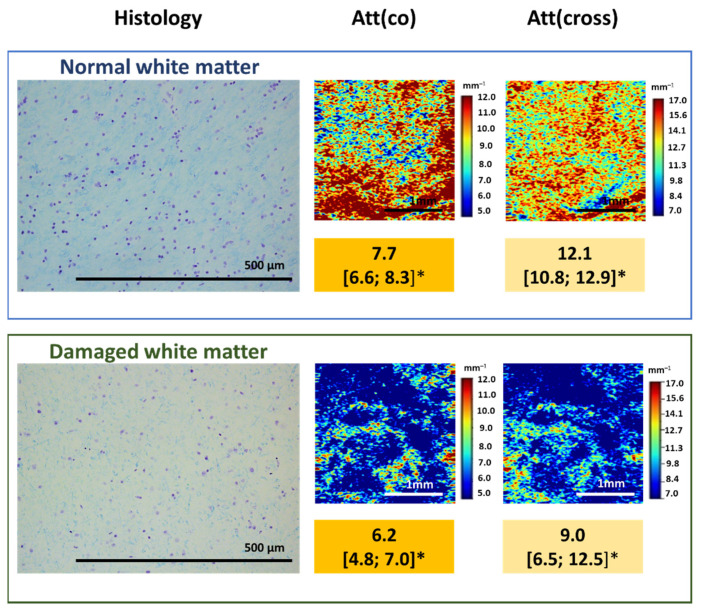
Damage to myelinated fibers in the region of interest causes the decrease in attenuation coefficient values both in co- (Att(co)) and cross-polarizations (Att(cross)), which is reflected on color-coded optical maps—the bright colors (red, orange) of myelinated fibers change to pale blue of damaged myelin. * Me [Q1; Q3]–Me–median value; Q1 and Q3 are the values of the 25th and 75th percentile of the distribution.

As was already mentioned, myelin is the main component that affects the OCT signal attenuation when white matter is studied. This relationship can be used to assess the degree of damage to myelinated fibers in the studied area, their density and arrangement. Our pilot studies of the relationship between the nature of the OCT signal and the state of the white matter in the peritumoral area demonstrated that damage to myelinated fibers due to tumor invasion leads to a change in the attenuation of the OCT signal, which can be assessed both qualitatively and quantitatively. At the same time, a slowdown in the signal attenuation in both polarizations is observed, which leads to a decrease in the values of the calculated attenuation coefficients, which, in turn, is reflected in the optical maps (Figure 5).

Despite the prospects of using OCT for assessing myelin fibers state, further research is needed to establish the exact relationship between the morphological and optical properties of white matter of the brain. 

## 8. OCT for Stereotactic Biopsy

Currently stereotactic biopsies are a routine neurosurgical procedure for the diagnosis of glial brain tumors and intracranial lymphomas. However, there is a risk of acquiring non-diagnostic samples from outside the viable tumor volume (such as necrotic/gliotic tissue or normal white matter), which suggested repeated neurosurgical interventions and has been reported in up to 24% of stereotactic biopsy series [80,81,82]. Therefore, the serial biopsies [83,84] and intraoperative neuropathological assessment [82,83,85] are commonly applied to improve the diagnostic yield and accuracy of stereotactic biopsies. These techniques, however, are associated with major drawbacks: (1) intraoperative neuropathological assessment is time consuming, costly, and generally not permanently available [82,86,87]; (2) acquisition of serial biopsies is associated with an increased risk of intracranial hemorrhages, which have been reported in 0.3–59.8% of cases [81,88,89] and contribute considerably to the reported morbidity of 0–16.1% [82,83,90,91] and mortality (3.9%) [81,82,90,91]. 

**Figure 6:**
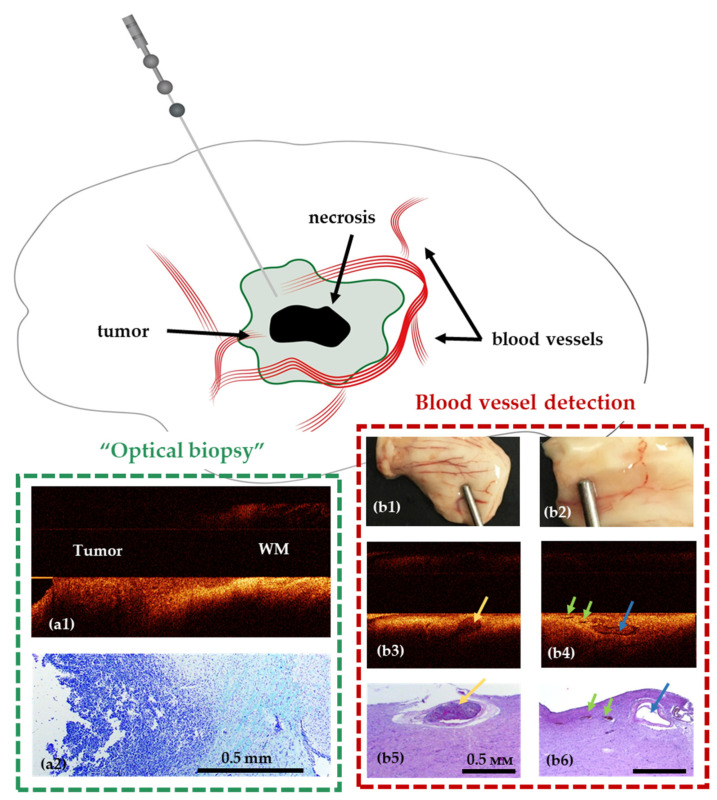
Use of stereotactic OCT needle for identification of tumor within white matter (**a1**,**a2**) and blood vessels (**b1**–**b6**) in preclinical studies. Visualization of white matter (WM)—tumor border using OCT in a model of glioblastoma 101.8 grafted into the rat brain and surrounding brain structures (frontal section) and corresponding histology (**a2**). OCT visualization of blood vessels in normal human brain samples (post mortem samples); (**b1**,**b2**)—digital photographs of the samples; (**b3**,**b4**)—OCT images obtained using a needle system; (**b5**,**b6**)—corresponding histological images. The width of the lumen of blood vessels, measured on histological preparations: 650 μm (**b3**), 300 μm (blue arrow) and 125–150 μm (green arrows) (**b4**).

Recently suggested stereotactic OCT probes for intraoperative optical biopsy and blood vessel detection allow enhancing the accuracy and safety of the procedure by solving the following problems—visualization of blood vessels in the biopsy area and identification of tumorous tissue [92] (Figure 6). This will allow avoiding intraoperative histopathological examination of tissue and avoiding the occurrence of intracerebral hemorrhages. The OCT probe can be integrated into a standard biopsy needle, giving the opportunity to monitor the movement of the needle towards the target, as well as to perform direct analysis of the tissue in the biopsy area. The stereotactic probe proposed by McLaughlin et al. was considered for detection of vessels in the area of sampling [93,94,95].

## 9. The Place of OCT among Other Intraoperative Imaging Techniques

Currently, methods of biomedical optics are most promising in the different areas of neurosurgery and especially in the field of brain tumor surgery [96,97]. They can have potential to make procedures safer and more accurate, and might improve the extent of resection, offering direct visualization of the tumor with cellular resolution microscopic OCT using high numerical aperture (NA). The main goal of using these methods is to detect tumor cells infiltration of brain tissue; also, they can be thought of as methods of OCT-based target biopsy during stereotactic procedures. The results of the current investigation show the perspectives of using confocal microscopy [98,99,100], Raman spectroscopy [101,102,103], fluorescence lifetime spectroscopy and imaging [104,105,106,107] and OCT [20,24,108]. Providing high-resolution imaging optical technologies may be regarded as substitution or, more likely, supplementation of traditional imaging such as MRI, ultrasound (US), 5-ALA or other fluorescence agents [96,97,109]. These methods have already demonstrated the necessity of their application for achieving maximal safe resection in glioma surgery. However, the unique limitations of these methods can necessitate the additional high-resolution assessment of tissue in the cases of conflicting or controversial results. The comparative characteristics of traditional and microscopic technologies in surgical guidance of gliomas are summarized in Table 2.

Most technologies have several strong limitations to being a standalone technology: (1) small penetration depth; (2) limited field of view; (3) the large size of used devices, which is not adapted for operation room workflow. Considering the wide spectrum of currently suggested optical technologies it must be mentioned that OCT has already been adapted for operation room workflow by integration in surgical microscope [20,21] or using various optical probes [22,23]. Moreover, the combination of OCT and a robotic driven surgical microscope in an experimental setting has allowed full-field OCT scanning [44]. 

Hartmann et al. have described the microanatomical structures such as the subarachnoid space, inlying blood vessels or the brain cortex using full-field microscope-integrated OCT [21,79]. OCT could increase security during certain critical surgical steps by delineation of crucial structures during tumor resection at major venous blood vessels, transsulcal preparation and dissection of the Sylvian fissure [27].

Among the wide spectrum of optical technologies, there is an increase of resolution ability of the scanning area and the ability of imaging tissue’s structure (e.g., blood vessel) decreases. The advantage of OCT is the ability of simultaneous visualization, both tissue structure visualization and detection of optical properties. It is also important that OCT does not need to use contrast agents. For blood vessels detection and safety of stereotactic procedures, some methods were considered, but they do not provide an OCT-based target biopsy option [110]. For simultaneous visualization of blood vessels and detection of tissue type, the endoscopic probe using 5-ALA can be used [111], but using 5-ALA has its limitations in low-grade tumors.

## 10. OCT Future Perspectives in Glioma Surgery

The following directions for the development of OCT in brain tumor surgery can be suggested: 

(1) The integration of different OCT modalities with a large field of view in superposition with traditional imaging methods as a multimodal approach has been considered for future brain tumor surgery. High-speed MHz-OCT has already been demonstrated to completely scan large tissue volumes with very high density in about one second, or with reduced resolution real-time videorate display [112,113]. The recent adaptation of a MHz-OCT to an operational microscope for neurosurgery allows the display of the resection volume in real time and can be augmented with OPM white light and fluorescence images. 

(2) In situ endoscopic microscopic OCT probes with high numerical aperture (NA) in order to achieve subcellular resolution in contact with the tissue will be a notable step towards optical in vivo biopsy and can support extracting appropriate biopsies and guiding the resection. In particular, the newly developed dynamic microscopic OCT reveals that cellular and extracellular metabolic activity has the potential for tumor discrimination either in situ or directly after biopsy extraction [114,115].

(3) Theranostic implementation of OCT as a part of an intelligent optical diagnosis and treatment system for tumor removal or tumor ablation procedures, with automated vessel localization. Laser coagulation and ablation by modern fiber-based systems in the 2 µm spectral range offer high ablation rates for tissue dissection and sufficient closure rate of microvessels during resection. This accelerates the surgery by making frequent instrumental change to bipolar scissors unnecessary and decreasing the rate of adverse tissue coagulation.

The implementation of machine learning and artificial intelligence methods for the classification of the received OCT images could simplify and improve accuracy of tumor detection during resection. With regard to visualization of brain tissue, works that demonstrate the methodological part of a set of images, their processing and histological assessment for subsequent use in training the classifier algorithm have been published, which can subsequently be used for navigation directly during tumor resection [116].

Juarez-Chambi et al. presented work on the application of artificial intelligence for automatic in situ detection of glioma infiltration in real time with high values of sensitivity and specificity for the detection of tumor-infiltrated tissue (sensitivity > 90%; specificity > 82%) [23]. Using this approach, real-time neurosurgical guidance can be provided with a three-dimensional color-coded map of the studied tissue site (Figure 2).

The use of artificial intelligence methods supports the development of combined OCT-laser ablation systems based on providing real-time tumor identification during the ablation process [117,118,119,120,121,122]. However, the integration of OCT and laser ablation has met the same dilemmas, including the challenges of use of the attenuation for tumor detection due to changes in optical properties after laser ablation. Additional studies and engineering development are required to assess the implications of the presented results for clinical neurosurgery.

## 11. Conclusions

In summary, OCT has demonstrated some advantages and has certain potential in ultrahigh-resolution brain imaging and neurosurgical guidance during brain tumor resection or stereotactic biopsy. OCT imaging has several benefits in the field of intraoperative technologies in neurosurgery: high resolution, high speed, low cost, label free, non-invasiveness and convenient performance. OCT has demonstrated, up to now only in small experimental settings, high sensitivity and specificity in distinguishing white matter and tumorous tissue using the criteria for visual assessment of OCT images and quantitative analysis by application of artificial intelligence. However, the diagnostic accuracy of OCT for identification of tumor infiltration within the grey matter is still unclear and further investigations are needed. 

Nevertheless, OCT has the potential to make procedures more safe and accurate; the right and effective implementation of OCT into the operation room workflow should be discussed. The current OCT devices do not provide wide-field imaging and therefore independent use of OCT during brain tumor resection can be difficult. OCT can be used in combination with wide-field imaging modalities such as MRI, US or fluorescence in controversial situations where the detection of microscopic tumor infiltration with high specificity and sensitivity will be useful. 

The theranostic implementation of OCT as a part of intelligent optical diagnosis and automated lesion localization and ablation could reach a precision which allows the use of this technology in automation or as an intelligent feedback system in future tumor surgery. This future implementation of OCT looks promising in translational medicine.

## Figures and Tables

**Table 1:** Sensitivity and specificity of OCT in distinguishing tumorous and non-tumorous tissues.

Study	Type of Study	Study Population	Tissue Type	The Type of Assessment	Sensitivity/Specificity
Böhringer et al., 2009 [20]	In vivo	9 patients (Grade 2–4)	Cortex White matter Tumor	qualitative, quantitative	no data, correlation between the scoring of the optical tissue analysis and the result of the histology (χ^2^ test; r = 0.99).
Kut et al., 2015 [24]	In vivo (mice) Ex vivo (human)	in vivo—5 mice ex vivo—32 glioma patients (Grade 2–4)	Cortex White matter Tumor	quantitative color-coded maps	100/80% for LGG 92/100% for HGG
Yashin et al., 2019 [46]	Ex vivo (human)	30 glioma patients (Grade 2–4)	Cortex White matter Tumor	quantitative color-coded maps	95.6–90.1/81.3–87.5% for all tumors 100/100% for tumor without necrosis 91.5–81/81–87.5% for tumor with necrosis
Yashin et al., 2019 [22]	Ex vivo (human) In vivo (human)	ex vivo—30 glioma patients (Grade 2–4) in vivo—17 glioma patients (Grade 2–4)	Cortex White matter Tumor	qualitative	82–85/92–94% for LGG/HGG
Almasian et al., 2019 [76]	In vivo (human)	5 patients (Grade 2–4)	Cortex White matter Tumor	quantitative	100/80% for LGG 92/100% for HGG,
Juarez-Chambi et al., 2019 [23]	Ex vivo (human)	9 patients (Grade 2–4)	Cortex White matter Tumor	quantitative color-coded maps	90.16/80.95% for LGG 95.45/82.14% for HGG 90.55/82.73% for LGG/HGG

LGG—low-grade gliomas (Grade 1–2), HGG—high-grade gliomas (Grade 3–4).

**Table 2:** Comparison of different technologies in surgical guidance of gliomas.

	iMRI	ioUS	5-ALA	Raman	Confocal Microscopy	OCT
Contrast physics	Nuclear magnetic resonance	Sound	Backscattering 5-ALA fluorescence	Raman scattering	Backscattering fluorescence	Backscattering
Resolution	3–20 mm^3^	0.3 mm^3^	0.0001 mm^2^	0.00000025 mm^2^	<0.001 mm^2^	0.004 mm^3^
Penetration	No limit	~80 mm	~300–800 µm	~1 mm	300–800 µm	≤2 mm
Field of scanning	Whole brain	12,500 mm^3^	75–2000 mm^2^	0.1225 mm^3^	~0.1 mm^2^	8–16 mm^3^
Real-time imaging and continuous guidance	No	Yes	Yes	Yes	Yes	Yes
Supports label-free	Yes	Yes	No	Yes	Limited	Yes
Numerical data	No	No	Yes	Yes	Yes	Yes
Surgical microscope integration	No	No	Yes	No	No	Yes
Sensitivity	75%	88–95% for HGG	82.6% for HGG	94% for HGG91% for LGG	85% for HGG90% for LGG	~90–100% for LGG~92–95% for HGG
Specificity	96–100%	62–98% for HGG	97.4% for HGG	91% for HGG91% for LGG	81% for HGG93% for LGG	~80–100% for LGG~90–100% for HGG
GTR achieving	38–100%	73.4%	~76%	No data	No data	No data

iMRI—intraoperative MRI; iUS—intraoperative ultrasound; LGG—low-grade gliomas (Grade 1–2), HGG—high-grade gliomas (Grade 3–4).
